# A Handsome Tribute to Merit

**Published:** 1848-01

**Authors:** 


We extract the following notice from one of the daily papers of this city. It relates to an under-graduate of Jefferson Medical College, who has shown by his example how much may be learned in the space of a few months, by one who energetically devotes himself to his studies.

A HANDSOME TRIBUTE TO MERIT.

At a meeting of the members of the Jefferson Medical Class of 1846–7, held on Monday, the 22d of November, Duncan Chambers, of Pennsylvania, having been called to the Chair, and Mr. A. J. Foard, of Georgia, appointed Secretary, the object of the meeting was stated, and an appropriate preamble and resolutions were read and unanimously adopted.

The object of the meeting, as stated, was to procure and present to George H. Howell, a member of the Jefferson Medical Class of 1846–7, a Case of Surgical Instruments, as a testimony of their esteem and approbation of the zeal and ability with which he had prosecuted his medical studies, and by which he had, in the short space of twenty-two months, qualified himself, as decided by the Navy Board of Examiners, for the honorable and responsible position of Assistant Surgeon in the Navy.

The presentation took place on Wednesday evening, November 24th, in the Medical Hall of Jefferson Medical College, in the presence of a large and highly respectable audience.

The presentation was made to Dr. Howell by Mr. D. Chambers, in a neat and appropriate address, in which he took occasion to express, in behalf of the donors, the admiration with which they regarded his talents and medical attainments, and the strong assurance they felt that his future career would equal in success and brilliancy that which had already won the highest regard and esteem of his Professors and class-mates. To which Dr. Howell responded in a manner that showed that modesty and true merit are twin sisters. To say that his language evidently conveyed the real sentiments of the heart—is to give to those who know him best, a correct idea of the character of his remarks.

